# Tissue Engineering Construct for Articular Cartilage Restoration with Stromal Cells from Synovium vs. Dental Pulp—A Pre-Clinical Study

**DOI:** 10.3390/pharmaceutics16121558

**Published:** 2024-12-05

**Authors:** Tiago Lazzaretti Fernandes, João Paulo Cortez Santanna, Rafaella Rogatto de Faria, Enzo Radaic Pastore, Daniela Franco Bueno, Arnaldo José Hernandez

**Affiliations:** 1 Sports Medicine Division, Institute of Orthopedics and Traumatology, Hospital das Clínicas HCFMUSP, Faculdade de Medicina, Universidade de São Paulo, São Paulo 05403-010, SP, Brazil; joaopcortez@hotmail.com (J.P.C.S.); rogatto.rafaella@gmail.com (R.R.d.F.); pastore.enzo@gmail.com (E.R.P.); ajhernandez@uol.com.br (A.J.H.); 2 Hospital Sírio-Libanês, São Paulo 05403-010, SP, Brazil; 3 Faculdade Israelita de Ciências da Saúde Albert Einstein, São Paulo 05403-010, SP, Brazil; dbuenousp@gmail.com

**Keywords:** tissue engineering, mesenchymal stromal cells, dental pulp, synovium, cartilage injuries, cartilage restoration

## Abstract

Background/Objectives: Cartilage injuries and osteoarthritis are prevalent public health problems, due to their disabling nature and economic impact. Mesenchymal stromal cells (MSCs) isolated from different tissues have the immunomodulatory capacity to regulate local joint environment. This translational study aims to compare cartilage restoration from MSCs from the synovial membrane (SM) and dental pulp (DP) by a tissue-engineered construct with Good Manufacturing Practices. Methods: A controlled experimental study was conducted on fourteen miniature pigs, using scaffold-free Tissue Engineering Constructs (TECs) from DP and SM MSCs, with a 6-month follow-up. Total thickness cartilage defects were created in both hind knees; one side was left untreated and the other received a TEC from either DP (n = 7) or SM (n = 7). An MRI assessed the morphology using the MOCART scoring system, T2 mapping evaluated water, and collagen fiber composition, and histological analysis was performed using the ICRS-2 score. Results: The untreated group had a mean MOCART value of 46.2 ± 13.4, while the SM-treated group was 65.7 ± 15.5 (*p* < 0.05) and the DP-treated group was 59.0 ± 7.9 (n.s.). The T2 mapping indicated a mean value of T2 of 54.9 ± 1.9 for native cartilage, with the untreated group at 50.9 ± 2.4 (*p* < 0.05). No difference was found between the T2 value of native cartilage and the treated groups. The ICRS-2 mean values were 42.1 ± 14.8 for the untreated group, 64.3 ± 19.0 for SM (*p* < 0.05), and 54.3 ± 12.2 for DP (n.s.). Conclusion: MRI and histological analysis indicated that TEC treatment led to superior cartilage coverage and quality compared to the defect group. TECs from SM demonstrated better results than the defect group in the histological assessment.

## 1. Introduction

Cartilage injuries and osteoarthritis are prevalent issues that pose significant public health challenges, leading to considerable disability and economic burdens on healthcare systems, particularly as the elderly population grows [1]. Cartilage defects can cause several complications for the individual, such as changes in the biomechanics and homeostasis of the joint, damage to the adjacent subchondral bone, decreased mobility, degeneration, and knee osteoarthritis directly affecting the quality of life. Thus, the study of new therapies for cartilage lesions is of high clinical relevance [2,3].

In recent decades, tissue engineering has emerged as a multidisciplinary field, and central to this approach is the use of mesenchymal stromal cells (MSCs), which have gained significant attention due to their ease of collection, capacity for cell proliferation and differentiation, and immunomodulatory properties that help regulate the local environment of the articular joint. These cells can be isolated from various tissues, including adipose tissue, bone marrow, synovial membrane, and dental pulp [4,5]. However, dental pulp stromal cells (DPSCs) and synovial membrane stromal cells (SMSCs) are alternatives that can be obtained from procedures the patient would already undergo, such as routine arthroscopy for SMSCs or from less invasive procedures which can be performed by a dentist for DPSCs.

Bueno et al., 2018 [6] showed that DPSCs can differentiate into chondroblasts, making them promising candidates for treating cartilage injuries, and demonstrate greater proliferative and immunomodulatory capacity in comparison to bone marrow MSCs [6]. Fernandes et al., 2018 [7] demonstrated that SMSCs exhibit high chondrogenic potential and can be harvested with minimal complications [7,8]. 

Furthermore, when it comes to local cell delivery, a scaffold-free technology known as a tissue engineering construct (TEC) has been considered as a potential delivery system [5]. Therefore, the hypothesis of the study is that treatment with a TEC offers superior outcomes compared to the conventional method of preparing cartilage defects and disrupting the calcified cartilage layer.

This translational study with medium-sized pigs aims to compare cartilage restoration using MSCs derived from both the synovial membrane (SM) and dental pulp (DP), by tissue engineered treatment using the Good Manufacturing Practices techniques [9]. 

Nowadays, there is a gap of current therapies or active pharmaceutical ingredients for osteoarthritis treatment. Tissue engineering can promote the repair of chondral injuries and is dependent on selecting appropriate cells. In this article, histological and image evaluation were applied to compare the cartilage restoration by tissue engineering and cell therapy from mesenchymal stromal cells derived from the synovial membrane and dental pulp. Mesenchymal stromal cells can be collected through outpatient procedures performed by dentists, or through standard surgical procedures that are already part of the patient’s routine care. The impact of this study is to determine the optimal harvesting method for obtaining these stromal cells to advance tissue engineering for cartilage repair in humans.

## 2. Methods

### 2.1. Experimental Design

This controlled experimental study involved 14 Brazilian miniature pigs, all of which had a skeletal age indicative of sexual maturity ranging from 8 to 12 months and weighed between 19 and 22 kg (Minipig Pesquisa e Desenvolvimento, Ltda., Campina do Monte Alegre, SP, Brazil). A total of 28 surgeries were performed, considering the two posterior knees of each animal. Outcomes were assessed 6 months after surgery.

The project received approval from the Ethics and Scientific Committee of the Hospital das Clínicas, University of São Paulo (protocol: CAPPesq nº 15428, IOT nº 1216) and by the Ethics Committee on the Use of Animals of the Hospital Sírio Libanês (approval number: CEUA P 2017–05). All patients signed informed consent forms for dental pulp and synovial and fat-pad discarded tissue usage in the research [10].

### 2.2. Harvesting, Isolation, and Expansion of MSCs

MSCs were harvested from the synovial tissue of human knees and the dental pulp of deciduous teeth. 

The synovial tissue was collected from the knees of seven patients undergoing arthroscopic procedures for injuries related to the anterior cruciate ligament or meniscus. Exclusion criteria encompassed patients with a previous history of surgery, infection, or inflammatory arthritis, and pregnant women [7].

A sample weighing up to 1g was placed in a 50 mL Falcon tube filled with a 0.9% saline solution and was promptly sent to the Cell Processing Laboratory at Hospital Sírio Libanês (São Paulo, Brazil), where it was processed within six hours of collection [7]. Each sample was washed twice in a saline solution containing 4% penicillin-streptomycin. Digestion was carried out using 0.2% Collagenase NB 4G Proved Grade (Serva Electrophoresis, Heidelberg, Germany) for 90 min at 37 °C. The process was concluded by adding 4 mL of Dulbecco’s Modified Eagle Medium/Nutrient Mixture F-12 (DMEM/F-12; Gibco Invitrogen, Grand Island, NY, USA) supplemented with 15% fetal bovine serum (FBS; HyClone Cytiva, Wilmington, DE, USA) and centrifuging at 1500 rotations per minute for 5 min. The sample was then diluted in DMEM/F-12 with fetal bovine serum before plating the cells in a 25 cm^2^; cell culture flask.

The dental pulp was obtained from the deciduous teeth of seven healthy children (five boys and two girls, aged 7 to 9) who naturally lost their teeth, which were destined for disposal. The tissue was stored in a vial identified with patient data. The samples were added to a sterile collector with 2 mL of DMEM-F12 solution (Dulbecco’s Modified Eagle Medium/Nutrient Mixture F-12–Gibco Invitrogen, Grand Island, NY, USA) supplemented with 100 IU/mL of penicillin-streptomycin (Penicillin-Streptomycin; Gibco Invitrogen, Grand Island, NY, USA). The samples were also sent to the Cell Processing Laboratory at Hospital Sírio Libanês (São Paulo, Brazil) and processed on average 15 h after collection [9].

In the laboratory, dental pulp samples were rinsed twice in saline and digested with 1 mg/mL TrypLE (Gibco Invitrogen, Grand Island, NY, USA) in phosphate-buffered saline (PBS, pH 7.4) for 30 min at 37 °C. After digestion, samples were centrifuged for 5 min and then fragmented with a sterile scalpel. Each fragment was placed in a separate well of a 12-well plate and immersed in a basal medium consisting of DMEM/F-12 (Gibco Invitrogen, Grand Island, NY, USA) with 15% fetal bovine serum (FBS; HyClone Cytiva, Wilmington, DE, USA) and non-essential amino acids (MEM; Gibco Invitrogen, Grand Island, NY, USA). Samples were maintained at 37 °C with 5% CO_2_ in a humidified incubator.

The MSCs were cultured until they achieved 70% to 80% confluence of the entire area of the culture plate. Once this confluence was attained, the MSCs were expanded to reach the necessary quantity for the experiment [9]

The MSC samples were processed, cultured, and plated in accordance with Good Manufacturing Practice (GMP) techniques for human usage, following the directives elaborated by the national regulatory authority, ANVISA [9].

### 2.3. Characterization of MSCs

The characterization of SMSCs and DPSCs was conducted using flow cytometry on cells between their fourth and fifth passages. Specific monoclonal antibodies were applied, such as CD29-PE, CD44-PE, CD73-FITC, CD90-FITC, CD105-PE, CD166-PE, CD31-FITC, CD34-FITC, and CD45-PE (BD Biosciences, Franklin Lakes, NJ, USA). Analyses included an appropriate isotype-matched control antibody. Flow cytometry data were gathered using a FACSCalibur flow cytometer (BD Biosciences) and processed with Cell Quest Software version 3.3 (BD Biosciences, Franklin Lakes, NJ, USA) [7,9].

**Figure 1:**
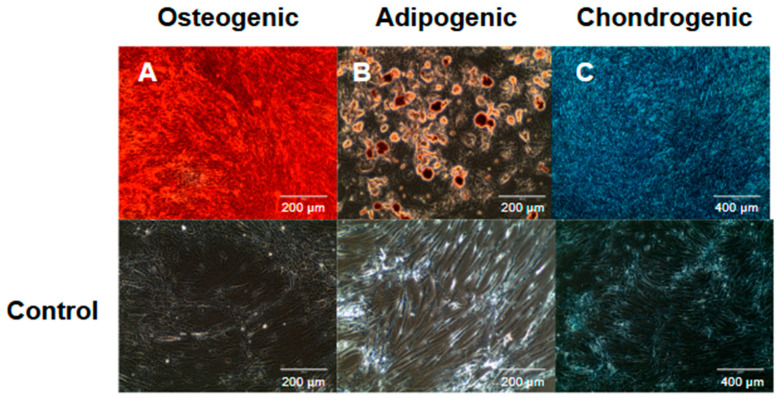
Cell differentiation: (**A**) Osteogenic differentiation visualized with Alizarin Red S staining (21 days), (**B**) adipogenic differentiation visualized with Oil Red O staining (18 days), and (**C**) chondrogenic differentiation visualized with Alcian Blue 8G staining (21 days), with respective controls shown below. Images acquired via optical microscopy (EVOS XL Cell Imaging System).

The MSC strains at passage four or five were induced in vitro to undergo osteogenic, chondrogenic, and adipogenic differentiation using the specific StemPro Differentiation Kits (Gibco Invitrogen, Grand Island, NY, USA) for osteogenesis, chondrogenesis, and adipogenesis. Media were prepared according to the manufacturer’s datasheets for each differentiation type. After a few days, staining was observed under an Olympus CK40 optical microscope (Figure 1) [10].

### 2.4. TEC Development

**Figure 2:**
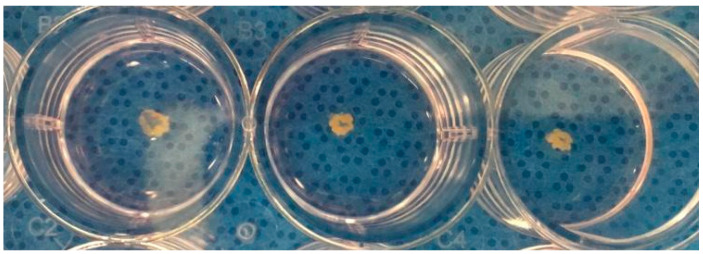
Three confluent tissue engineering construct samples after 15 days of culture forming a three-dimensional structure.

After cell culture, the SMSCs and DPSCs were plated on a 12-well culture dish at a density of 4.0 × 10^5^ cells/cm^2^ in a culture medium with 0.2 mM ascorbic acid 2-phosphate (Asc-2P; Sigma-Aldrich, St. Louis, MO, USA). After approximately 15 days, the cell constructs and extracellular matrix synthesized by themselves were detached from the substrate by applying shear stress using a pipette. The separate constructs were left in suspension to form a three-dimensional structure by active tissue contraction. This tissue was called a tissue engineering construct, TEC, and was composed of cells and an extracellular matrix (Figure 2) [5].

### 2.5. Animal Model and Surgical Technique

This study utilized 14 female miniature pigs, identified as BR-1. The animals were adult females and had an average weight of 28.5 kg and a standard deviation (SD) of 2.6. The average age of the animals at the time of surgery was 11.2 months, with a SD of 0.8. [10].

Data concerning the animals’ characteristics, care, and procedures were gathered in accordance with the ARRIVE Guidelines Checklist [11] and were stored in a digital repository called REDCAP [12].

The animals were induced for general anesthesia and placed on the operating table. At the time of surgery and in a randomized manner, the surgeon was informed which side would receive the TEC, and which would only have a defect [10].

**Figure 3:**
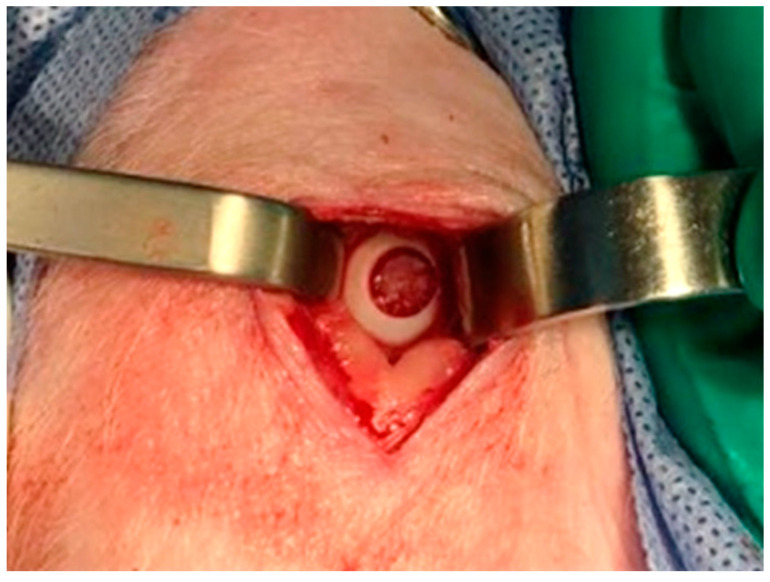
Full-thickness cartilage defect measuring 6 mm in the medial femoral condyle of the right knee’s hind limb.

A full-thickness cartilage defect, with a diameter of 6 mm (Figure 3), was created in the weight-bearing region of the medial femoral condyle on both hind limbs of each pig. Subsequently, the TEC was inserted into the defect [13].

In the control group, a treatment commonly used in surgical procedures to promote cartilage regeneration was performed. This approach involved preserving the native cartilage at a 90-degree orientation and curettage of the calcified cartilage layer to stimulate localized bleeding and fibroblast tissue formation. This protocol is frequently proposed in the literature for the treatment of cartilage defects.

The animals were treated post-operatively. At 6 months after surgery, the animals were euthanized and the hind limbs were disarticulated [10].

### 2.6. Evaluation Methods

#### 2.6.1. Magnetic Resonance Imaging

Imaging was conducted using a 7 Tesla high-field magnetic resonance imaging (MRI) scanner (Magnetom 7 Tesla, Siemens Healthcare, Forchheim, Germany), equipped with a head coil featuring 1 transmission channel and 32 receiving channels (Nova Medical, Inc. Wilmington, MA, USA), at the PISA Project (Faculdade de Medicina USP, São Paulo, Brazil). Images were acquired from both knees of the 14 pigs using two distinct sequences [14,15].

The first sequence performed was the 3D double echo steady state (3D-DESS) for morphological evaluation. The parameters applied were the following: repetition time (TR) = 12.2 ms, echo time (TE) = 4.1 ms, fractional anisotropy (FA) = 25º, voxel = 0.4 × 0.4 × 0.4 mm^3^, field-of-view (FoV) = 192 × 256 mm, slice thickness 0.4 mm, acquisition time 10:52 min) [14,15].

For these acquired images, sagittal and coronal views were used and articular cartilage repair tissue was evaluated using Magnetic Resonance Observation of Cartilage Repair Tissue (MOCART) 3D score. The score uses 11 categories to classify cartilage repair, ranging from 0 (no repair) to 100 points (complete repair of the cartilage defect).

The second sequence was the spin-echo with multi-echo to evaluate the water and collagen fiber composition of the cartilage based on T2 mapping creation using ImageJ software version 1.53 (National Institutes of Health). The parameters were the following: TR = 10,000 ms, 18 echoes, TE = 9/18/27/336/45/54/63/72/81/90/99/108/117/126/135/144/153/162; voxel = 0.6 × 0.6 × 2.0 mm^3^, FoV 93 × 229 mm, slice thickness 2.0 mm, acquisition time 18:44 min) [16,17].

For these acquired images, three consecutive sagittal sections were selected from each knee. In each section, one area of interest was selected representing the cartilage defect untreated and treated with the implantation of the TEC, and another was selected representing the intact chondral tissue (adjacent cartilage). After selecting the areas, the average T2 value was measured in each section of each knee, considering the native cartilage, groups treated with MSCs from SM and DP, and untreated groups [16,17].

Another assessment was carried out using the mean T2 value measurement of the deep and superficial areas of the previously selected areas of interest [17].

#### 2.6.2. Histological Evaluation

After MRI evaluation, both knees were dissected and subjected to histological evaluation in order to analyze the quality of the cartilage repair. A block around the defect measuring approximately 1.5 × 1.5 × 1.5 cm was cut and the tissue was fixed in 4% paraformaldehyde (PFA) and decalcified with ethylenediamine tetra-acetic acid (EDTA). The sections were prepared with a thickness of 4 μm and stained with hematoxylin and eosin (HE). Two sections from each animal were stained with toluidine blue to evaluate the color concentration of the extracellular matrix [18].

The ICRS-2 scoring system (Table 1) was used to assess the articular cartilage repair tissue and assigned a score for each of the 14 categories evaluated in the system, from 0 (worst result) to 100 points (best result). This evaluation was performed on the defect-only, SM treatment and DP treatment groups.

### 2.7. Statistical Analysis

Quantitative variables with normal distribution were described by measures of central tendency and dispersion (mean and standard deviation).

ANOVA analyses followed by post hoc Bonferroni were used to compare the values obtained from the MOCART 3D system score and the ICRS-2 score. This test was used as the knees compared were from the same pigs and were subjected to the same external stimuli.

ANOVA analyses were also used to compare the mean T2 values of the areas of interest, chondral defect and adjacent cartilage, and the T2 values from the deep and superficial regions in each area. 

The correlation between the different scoring systems (MOCART and ICRS-2) was measured with the Pearson correlation coefficient. The Software Sigmaplot version 15.0 (Systat Software, Inc., San Jose, CA, USA) was used in the calculations. The level of statistical significance adopted was equal to 5%; that is, the test results were considered statistically significant when *p* < 0.05.

## 3. Results

### 3.1. Characterization of MSC Strains

**Figure 4:**
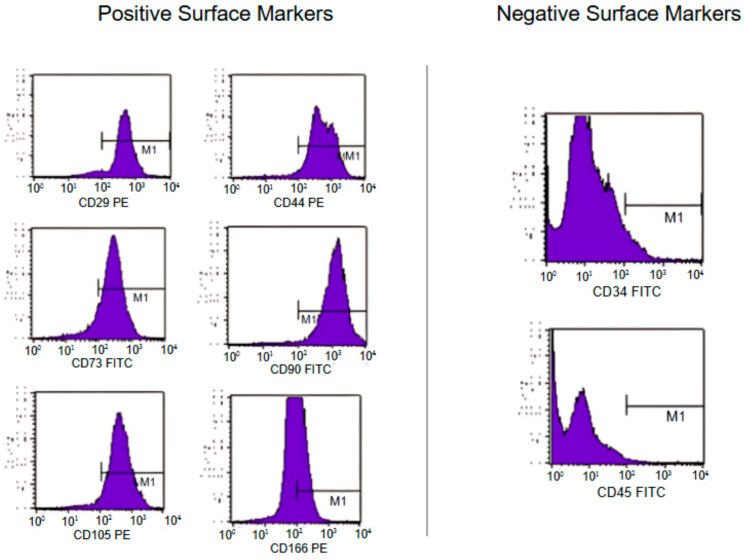
Flow cytometry analysis showing positive reactions to mesenchymal markers (CD29, CD73, CD105, CD44, CD90, and CD166) and negative reactions to hematopoietic (CD34 and CD45). In purple, the cell population that presents the respective marker.

The cell characterization by flow cytometry and in vitro induction confirmed the multipotentiality of cells derived from the SM and DP, since they differentiated into osteogenic, chondrogenic, and adipogenic strains. In addition, MSCs showed positive reactions for mesenchymal markers (CD29, CD44, CD73, CD105, CD90, and CD166) and negative reactions to hematopoietic (CD34 and CD45) and endothelial markers (CD31) (Figure 4 and Table 2). 

### 3.2. Macroscopic Characterization

A macroscopic evaluation was conducted to visually assess the repair quality of the cartilage defect, focusing on surface appearance, tissue integration, and color match with surrounding cartilage.

**Figure 5:**
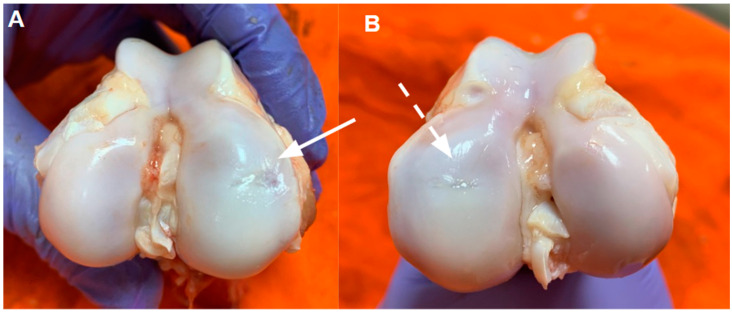
Cartilage defect 6 months after surgery, arrows indicate the region where the defect was made. (**A**) Defect-only (solid arrow); (**B**) defect treated with a tissue engineering construct (TEC) (dashed arrow).

As observed in Figure 5, untreated cartilage defects displayed limited tissue coverage (Figure 5A). In contrast, cartilage defects treated with a TEC seeded with MSCs demonstrated nearly complete coverage of the defect (Figure 5B).

### 3.3. Magnetic Resonance Imaging

**Figure 6:**
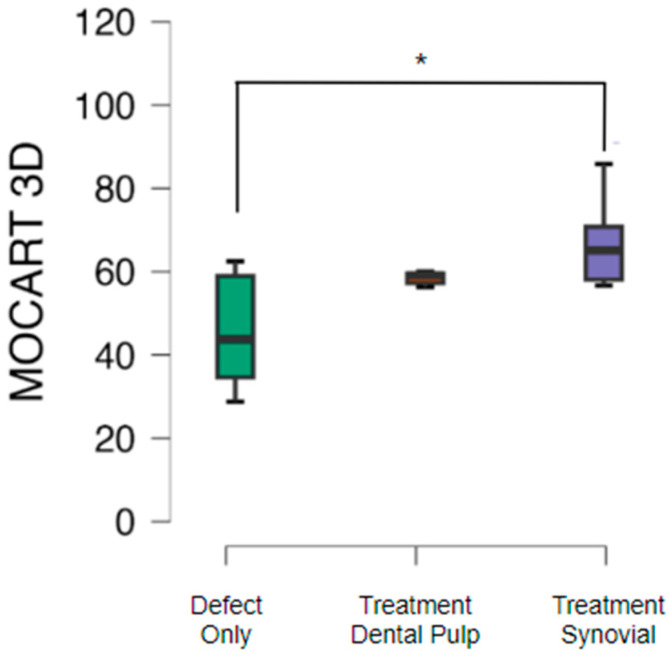
Overall assessment score values in MOCART 3D score for the defect-only (untreated), dental pulp treatment, and synovial treatment groups, showing a significant difference * (*p* < 0.05) between the defect-only group and the group treated with a TEC from the synovial.

Morphological assessment of cartilage repair with the MOCART 3D score showed that cartilage repair in knees subjected only to the cartilage defect presented a mean MOCART value of 46.2 with a standard deviation of 13.4. The group treated with a TEC from SM had a mean MOCART value of 65.7 with a standard deviation of 15.5 (*p* < 0.05), while the mean value obtained was 59.0 with a standard deviation of 7.9 with a TEC from the DP (Figure 6). 

**Figure 7:**
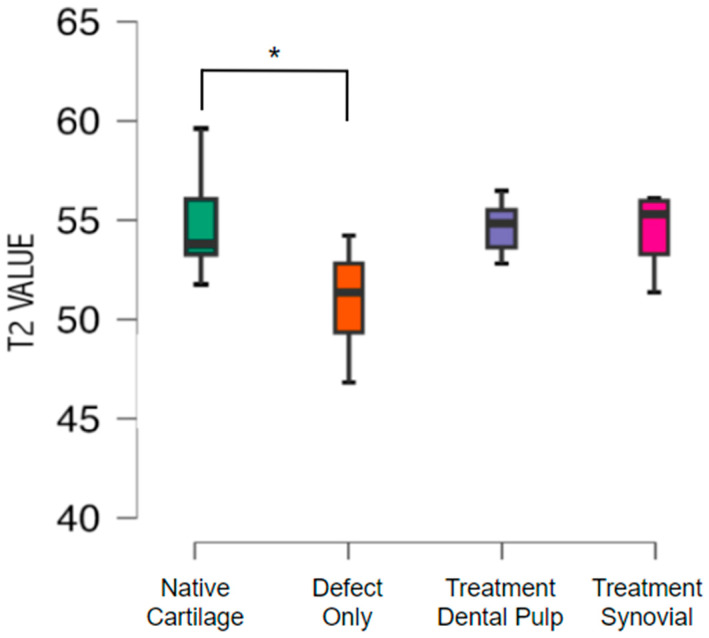
Mean T2 values of the groups from native (adjacent cartilage), defect-only (untreated), dental pulp treatment, and synovial treatment groups. There was a significant difference * (*p* < 0.05) between native cartilage and defect-only groups.

Cartilage composition was assessed with T2 mapping, showing a mean value of T2 of 54.9 with a standard deviation of 1.9 in the native cartilage. The untreated group exhibited a mean T2 value of 50.9 with a standard deviation 2.4 (*p* < 0.05). No difference was found between the native cartilage and the treated groups. The mean T2 value from the group treated with a TEC from the SM was 54.31 with a standard deviation of 2.07, and from the DP was 54.54 with a standard deviation of 1.47 (Figure 7)**.**

When measuring the T2 value by zones of the native cartilage and in the groups that received treatment with a TEC of the PD and SM, there was a decrease comparing the value of the superficial zone and the value of the deep zone. On the other hand, when analyzing the defect group, there was a small increase from the superficial zone to the deep zone, with no significant difference.

The T2 value (mean ± standard deviation) obtained in the superficial zone of the native cartilage (n = 12) was 59.3 ± 2.4 and in the deep zone was 50.7 ± 2.9 (*p* < 0.001). Considering the DP group (n = 6), the mean T2 value was 57.5 ± 2.7 for the superficial zone and 51.6 ± 2.0 for the deep zone (*p* < 0.05). For the SM group (n = 6), the mean T2 value was 57.1 ± 3.9 for the superficial zone and 51.5 ± 2.0 for the deep zone (*p* < 0.05). The defect group (n = 12) presented a mean T2 value of 50.5 ± 4.9 for the superficial zone and 51.4 ± 2.6 for the deep zone.

### 3.4. Histological Evaluation

**Figure 8:**
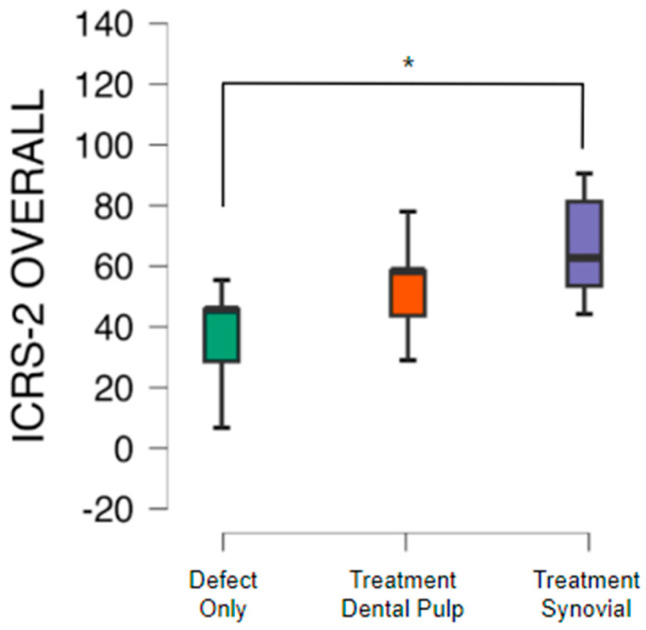
Mean value of overall assessment of cartilage repair using the ICRS-2 histological score for the defect-only (untreated), dental pulp treatment, and synovial treatment groups. A significant difference * (*p* < 0.05) was found between the defect-only group and the group treated with a TEC from synovial membrane.

**Figure 9:**
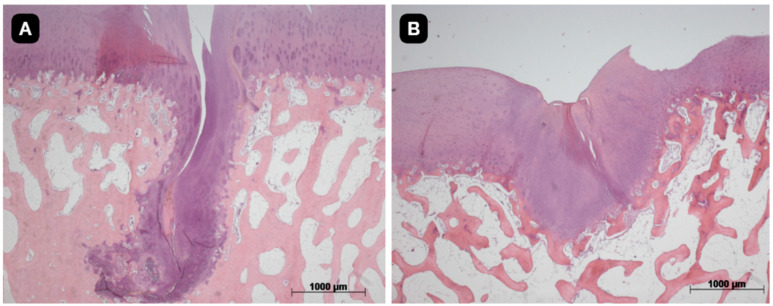
Histological evaluation of (**A**) defect-only in posterior knee; (**B**) defect treated with a TEC from synovial membrane in posterior knee.

**Figure 10:**
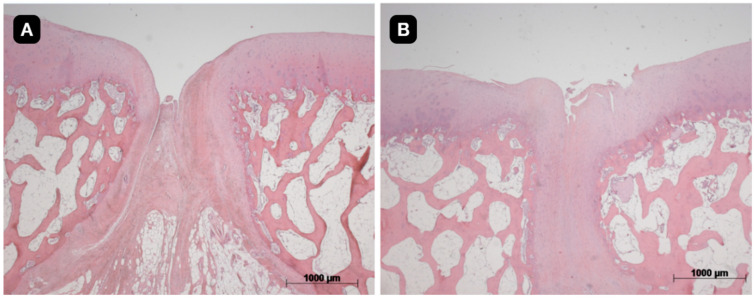
Histological evaluation of (**A**) defect-only in posterior knee; (**B**) defect treated with a TEC from dental pulp in posterior knee.

The quality of the tissue and its intrinsic characteristics were assessed by the ICRS-2 score system (Table 3). The untreated group presented a mean value 42.1 with a standard deviation of 14.8. The group with a TEC from SM had a significant difference in comparison to the untreated group and presented a mean value of 64.3 with a standard deviation of 19.0 (*p* < 0.05). The group with a TEC from DP presented a mean value of 54.3 with a standard deviation of 12.2 (Figure 8, Figure 9 and Figure 10).

## 4. Discussion

This preclinical study compares cartilage restoration using a scaffold-free tissue engineering construct (TEC) between SM- and DP-derived MSCs. The defect created in this study replicates a focal cartilage injury in the knee, and the miniature pig model closely resembles the human knee. This model is widely used in the literature, given its high relevance for clinical translation in treating knee cartilage defects [19]. It showed better results in the SM group compared to the control in MRI and histological analysis. Additionally, the SM group outperformed the dental pulp (DP) group in MRI assessments.

Mei et al., 2024 [20] highlight the immunosuppressive properties of MSCs, in agreement with what was proven by Li et al., 2009 [21] and other authors, in which human MSCs elicited no immune reactions in miniature pigs, supporting the viability of using heterogeneous cells between species. In addition, Ando et al., 2008 [5] and Shimomura et al. (2010) [22] first reported the feasibility of creating scaffold-free approaches for chondral repair in a swine model using MSCs from the SM. In contrast, the present study is known to be the first to use MSCs from DP and SM to create the TEC and to report the data from histological and imaging evaluation.

Magnetic resonance imaging (MRI) is a non-invasive tool for assessing hyaline cartilage quality, enabling the translation of animal model findings into clinical studies. A pilot study by Shimomura et al., 2023 [23] reported sustained improvements in clinical outcomes and MRI findings five years post-implantation of the TEC, with a mean MOCART 3D score (mean ± standard deviation) of 82.0 ± 13 in the group treated with a TEC. While this score decreased slightly from the two-year follow-up, it increased compared to the six-month follow-up, indicating ongoing efficacy. No control group was used [24]. 

In the present study, the TEC-treated group also showed significant improvements over the defect-only group, particularly with MSCs derived from the SM. However, the average value of the MOCART 3D score was lower, 65.7 from the SM and 59.0 from the DP, potentially due to the shorter six-month evaluation period compared to the five-year follow-up of Shimomura et al., 2023 [23].

MRI is sensitive to specific changes in the chemical composition and structure of cartilage even before severe morphological changes, and T2 mapping is a technique to assess this aspect and complement morphological evaluation [15,25,26]. In the current study, similar to Theruvath et al., 2021 [27] and Shimomura et al., 2018 [24], significant differences in T2 values were observed between the defect-only and healthy cartilage groups. Shimomura et al., 2018 [24] also demonstrated that T2 values change over time, correlating with the maturation of repair tissue at 6- and 24-weeks post-surgery. Additionally, in the present study, the mean T2 values between native cartilage and the tissue formed by TEC from both groups were comparable after 6 months, suggesting similar tissue compositions.

Variations in T2 values across different cartilage zones reflect compositional differences; higher T2 values in the superficial zone indicate increased water content. This pattern aligns with findings by White et al., 2006 [17] who noted higher T2 values in the superficial regions of equine cartilage, and the present study with groups from native cartilage and TEC treatment derived from DP and SM. Notably, Shimomura et al., 2018 [24] did not observe significant differences in T2 values across various zones, which contrasted with histological assessments. 

Histological analysis serves as a valuable complement to cartilage imaging evaluations, offering insights into the intrinsic characteristics and quality of the tissue [14]. Ando et al., 2007 [28] evaluated the histology based on the ICRS-1 in a swine model that received TEC from DP-derived MSCs, and similarly to the present study, the histological repair score in the intervention group was significantly better compared to the defect-only group. Similarly, Shimomura et al., 2018 [24] evaluated repair tissue histology using the ICRS-2 score in patients who underwent TEC treatment, reporting a mean score of 80.0 ± 11.0, although no control group was included.

In the current study, the average ICRS-2 score for the DP-treated group was 54.2 ± 16.1, and for the SM-treated group, it was 64.2 ± 19.0 (*p* < 0.05), significantly better than the defect group’s score of 42.1 ± 14.7. These differences may be attributed to the evaluation timeline, as the present study assessed tissue at six months post-surgery compared to the 48 weeks used by Shimomura et al., 2018 [24]. Additionally, Gardner et al., 2019 [29] investigated tissue engineering interventions and employed the ICRS-2 scoring system, noting no significant differences at six weeks post-surgery, but significant improvements observed at twelve weeks.

DP-derived MSCs have shown promise for various applications, particularly in treating bone conditions like alveolar clefts [6,9,30]. These cells are easy to collect and can be obtained with minimal donor site morbidity and iatrogenic damage. While some studies have explored their potential for cartilage repair, a comparative analysis of dental pulp-derived MSCs (DPSCs) and synovial membrane-derived MSCs (SMSCs) remains lacking for cartilage injuries and osteoarthritis use [31,32,33]. Additionally, Xu et al., 2023 [34] indicated that SMSCs enhance MRI indices and histological scores, and their use with scaffold-free constructs for cartilage repair is safe and effective, leading to reduced pain, improved function, and higher quality of life and clinical outcomes, which corroborates to the results obtained in this study [34].

One of the limitations of this study is the absence of male animal models. It was decided to use female animals in the research project as part of the strategy to characterize the presence of donor cells.

This translational study presented an active pharmaceutical ingredient derived from tissue engineering therapeutic option known as TEC, for a highly prevalent condition with a high impact on public health. As it does not require an external scaffold, it is safer and can reduce the costs of treating cartilage injuries. As future steps, phase I/II and “first in human” clinical trials may be carried out.

## 5. Conclusions

TEC derived from SM led to superior cartilage coverage and quality compared to the defect group in MRI and histological analysis. In the MRI assessment, both DP and SM groups showed better results in comparison to the defect group. In the histological assessment, TEC from SM demonstrated better results than the defect group and had no difference to the treatment with a TEC from the DP.

## Figures and Tables

**Table 1:** ICRS-2 scoring system for histological assessment of cartilage tissue repair. Adapted from Mainil-Varlet et al., 2010 [18].

Histological Parameter	Score
1. Tissue Morphology (viewed under polarized light)	0%: Full thickness collagen fibers 100%: Normal cartilage birefringence
2. Matrix staining (metachromasia)	0%: No staining 100%: Full metachromasia
3. Cell morphology	0%: No round/oval cells 100%: Mostly round/oval cells
4. Chondrocyte clustering (4 or more grouped cells)	0%: Present 100%: Absent
5. Surface architecture	0%: Delamination, or major irregularity 100%: Smooth surface
6. Basal integration	0%: No integration 100%: Complete integration
7. Formation of a tidemark	0%: No calcification front 100%: Tidemark
8. Subchondral bone abnormalities/marrow fibrosis	0%: Abnormal 100%: Normal marrow
9. Inflammation	0%: Present 100%: Absent
10. Abnormal calcification/ossification	0%: Present 100%: Absent
11. Vascularization (within the repaired tissue)	0%: Present 100%: Absent
12. Surface/superficial assessment	0%: Total loss or complete disruption 100%: Resembles intact articular cartilage
13. Mid/deep zone assessment	0%: Fibrous tissue 100%: Normal hyaline cartilage
14. Overall assessment	0%: Bad (fibrous tissue) 100%: Good (hyaline cartilage)

**Table 2:** Characterization of synovial membrane mesenchymal stromal cells with percentages of positive and negative surface markers.

Type	Surface Marker	Percentages
Positive	CD166	57.1%
Positive	CD105	95.1%
Positive	CD90	97.5%
Positive	CD73	83.3%
Positive	CD44	93.5%
Positive	CD29	93.0%
Negative	CD117	5.0%
Negative	CD31	4.8%
Negative	CD45	4.1%
Negative	CD34	4.8%

**Table 3:** ICRS-2 parameters from dental pulp, synovial membrane, and control groups.

ICRS-2 Parameters	Dental Pulp	Synovial	Control	*p*
1. Tissue morphology (viewed under polarized light)	55.7 ± 14	67.1 ± 17.0	39.3 ± 24	* *p* < 0.05
2. Matrix Staining (metachromasia)	68.3 ± 20.4	86.7 ± 8.2	59.2 ± 26.4	n.s.
3. Cell Morphology	43.3 ± 12.1	65.7 ± 25.1	47.9 ± 28.1	n.s.
4. Chondrocyte clustering	78.3 ± 11.7	90.7 ± 11.7	85.8 ± 12.4	n.s.
5. Surface architecture	65.7 ± 22.3	58.6 ± 25.5	43.8 ± 21	n.s.
6. Basal integration	100 ± 0	92.9 ± 18.9	62.9 ± 43.4	n.s.
7. Formation of a tidemark	55.8 ± 33.5	72.9 ± 34.5	60.8 ± 24.2	n.s.
8. Subchondral bone abnormalities/marrow fibrosis	36.7 ± 40.3	61.4 ± 37.2	43.5 ± 26.9	n.s.
9. Inflammation	83.3 ± 18.6	97.1 ± 7.6	88.8 ± 27.4	n.s.
10. Abnormal calcification/ossification	84.3 ± 35.4	100 ± 0	98.5 ± 5.5	n.s.
11. Vascularization (within the repaired tissue)	81.7 ± 21.4	91.4 ± 22.7	90.8 ± 27.5	n.s.
12. Surface/superficial assessment	60 ± 30	67.1 ± 21.4	44.3 ± 22.7	n.s.
13. Mid/deep zone assessment	35 ± 38.9	57.1 ± 33	40 ± 19.1	n.s.
14. Overall assessment	54.3 ± 12.2	64.3 ± 19	42.1 ± 14.8	* *p* < 0.05

Note: * control vs synovial membrane, post hoc Bonferroni; n.s. = non-significant.

## Data Availability

The data presented in this study are available on request from the corresponding author due to privacy reasons.
